# High engraftment capacity of frozen ready-to-use human fecal microbiota transplants assessed in germ-free mice

**DOI:** 10.1038/s41598-021-83638-7

**Published:** 2021-02-23

**Authors:** Magali Berland, Julie Cadiou, Florence Levenez, Nathalie Galleron, Benoît Quinquis, Florence Thirion, Franck Gauthier, Emmanuelle Le Chatelier, Florian Plaza Oñate, Carole Schwintner, Sylvie Rabot, Patricia Lepage, Dusko Ehrlich, Joël Doré, Catherine Juste

**Affiliations:** 1 Université Paris-Saclay, INRAE, MGP, 78350 Jouy-en-Josas, France; 2 Université Paris-Saclay, INRAE, AgroParisTech, Micalis Institute, 78350 Jouy-en-Josas, France; 3 MaaT Pharma, Pharmaceutical Development, 69007 Lyon, France

**Keywords:** Metagenomics, Microbiome, Microbiology

## Abstract

The number of indications for fecal microbiota transplantation is expected to rise, thus increasing the needs for production of readily available frozen or freeze-dried transplants. Using shotgun metagenomics, we investigated the capacity of two novel human fecal microbiota transplants prepared in maltodextrin-trehalose solutions (abbreviated MD and TR for maltodextrin:trehalose, 3:1, w/w, and trehalose:maltodextrin 3:1, w/w, respectively), to colonize a germ-free born mouse model. Gavage with frozen-thawed MD or TR suspensions gave the taxonomic profiles of mouse feces that best resembled those obtained with the fresh inoculum (Spearman correlations based on relative abundances of metagenomic species around 0.80 and 0.75 for MD and TR respectively), while engraftment capacity of defrosted NaCl transplants most diverged (Spearman correlations around 0.63). Engraftment of members of the family *Lachnospiraceae* and *Ruminoccocaceae* was the most challenging in all groups of mice, being improved with MD and TR transplants compared to NaCl, but still lower than with the fresh preparation. Improvement of engraftment of this important group in maintaining health represents a challenge that could benefit from further research on fecal microbiota transplant manufacturing.

## Introduction

Fecal microbiota transplantation (FMT) is recognized as the most successful strategy to cure patients from recurrent *Clostridioides difficile* infection (rCDI). Indeed, the guidelines of both the American College of Gastroenterology^1^ and the European Society for Microbiology and Infectious Disease^2^ over the last years, and more recently updates of the American IDSA-SHEA^3^ and the European FMT Working Group^4^, recommend FMT as a treatment for rCDI. Over the last two decades, molecular tools for the assessment of the intestinal microbiota have progressed tremendously, revealing that severe and persistent distortions of the intestinal microbiota composition (dysbiosis) do accompany many non-infectious diseases of modern societies^5–11^. Thus, FMT has been viewed as potentially bringing benefits in a number of clinical or environmental contexts where equilibrium of the gut microbiota is severely disrupted^12–15^. Similarly, autologous FMT (own microbiota stored temporarily to be given back to the patient) has been perceived as the most immediate means for restoring one’s own microbiota in any instance where severe alteration can be anticipated^16,17^. As a result, one can reasonably expect the number of indications for FMT to go up considerably, thus increasing the needs for research and production of viable, stabilized frozen or freeze-dried transplants. Indeed, frozen or freeze-dried storage is necessary for safety controls of allogenic transplants, while it is simply an inherent and necessary part of the protocol in autologous FMT. To meet this growing demand, FMT has evolved over the last decade from the use of fresh fecal material, typically suspended in oxic physiologic saline, to more standardized protocols using frozen preparations^18^. At the same time, a number of original reports attested to the efficacy of frozen transplants for combating rCDI^19,20^, and this was re-emphasized in a recent meta-analysis^21^.

The current standard for cryopreservation of stool suspensions intended for FMT is in oxic physiologic saline added with 10% glycerol^4^, a permeating cryoprotectant agent. However, glycerol is not suitable for downstream lyophilization as it leads to a sticky, insufficiently dried product^22^. In a recent work^23^, we emphasized the selection of cryoprotective agents that will allow lyophilization as an optimal means of progressing towards oral administration of encapsulated forms in a near future. We selected maltodextrin and trehalose in combination^23^ since the association of this polymer and a disaccharide is known to show synergistic stabilizing effects on freeze-dried micro-organisms^24–26^. Indeed, small disaccharides such as trehalose have a ‘water substitution’ effect, replacing water molecules to form hydrogen bonds with cell membrane phospholipids and proteins so that a solid pseudo-hydrate structure, named glassy structure, is formed in the dehydrated biomaterial^24^. Polymers such as maltodextrins, due to their stearic hindrance, have a lower water substitution effect, but they function as osmotically inactive bulking compounds that cause spacing of the cells and increase viscosity. This improves the stability of the glassy structure during prolonged storage at ambient temperature^24^, a clinically advantageous and low-cost solution. We therefore proposed two maltodextrin-trehalose formulations for the production of frozen, live, and ready-to-use transplants that meet the requirements of the pharmaceutical industry and can further be freeze-dried^23^. One of them (trehalose:maltodextrin 3:1, w/w) prioritized membrane protection while the other (maltodextrin:trehalose 3:1, w/w) prioritized high viscosity, bulking properties, both giving high performances in vitro. Indeed, we demonstrated that these novel formulations stored in a − 80 °C standard freezer and then rapidly thawed at 37 °C, or even lyophilized and stored at ambient temperature, retained the best revivification potential in vitro, as proven by phylotranscriptomic profiles, metabolomic fingerprints, and flow cytometry assays^23^.

Herein, using shotgun metagenomics, we substantiated the capacity of frozen-thawed liquid formulations of those novel human fecal microbiota transplants to colonize the intestine of germ-free born mice, compared to the gold standard fresh stool.

## Results

**Figure 1:**
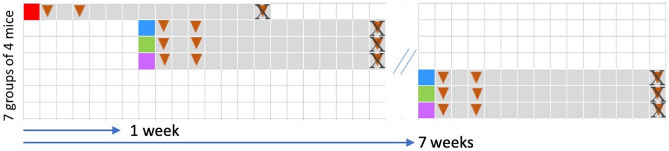
Experimental design. The first group of mice (n = 4) received a human stool suspension freshly prepared in NaCl (“red square”). The six following groups of mice (n = 4 per group) received the same stool specimen that has been prepared in NaCl (“blue square”) or MD (“green square”) or TR (“purple square”), and frozen for one or seven weeks. Fecal samples (“triangle”) were collected from each mouse at day 2, 4 and 15 after FMT for individual metagenomic profiling. End of experiment (“X”).

Our main purpose was to compare the engraftment capacity of three experimental frozen/thawed fecal transplants that have been prepared and frozen in one of the two novel diluents, MD (maltodextrin:trehalose, 3:1, w/w) or TR (trehalose:maltodextrin, 3:1, w/w), or simply in NaCl. All three experimental transplants were prepared from the same freshly voided fecal sample from a healthy adult human. We inoculated seven groups of four 8-weeks-old germ-free male mice, strain C57BL/6 J, fed γ-irradiated standard pellets. The control group received by gavage the fresh transplant suspended in NaCl; the six experimental groups received transplants prepared in either MD, or TR or NaCl and stored frozen at − 80 °C for either one or seven weeks (abbreviated forms W1 and W7) (Fig. 1). We based our analyses on shotgun metagenomic sequencing and binning of co-abundant genes into metagenomic species pangenome (MSP), a concept that allows precise detection and quantification of microbial species including those not cultivated to date^27^. This non-targeted, reference genome-independent approach is known to provide a broadened and deepened knowledge of complex environmental communities without any a priori consideration.

**Figure 2:**
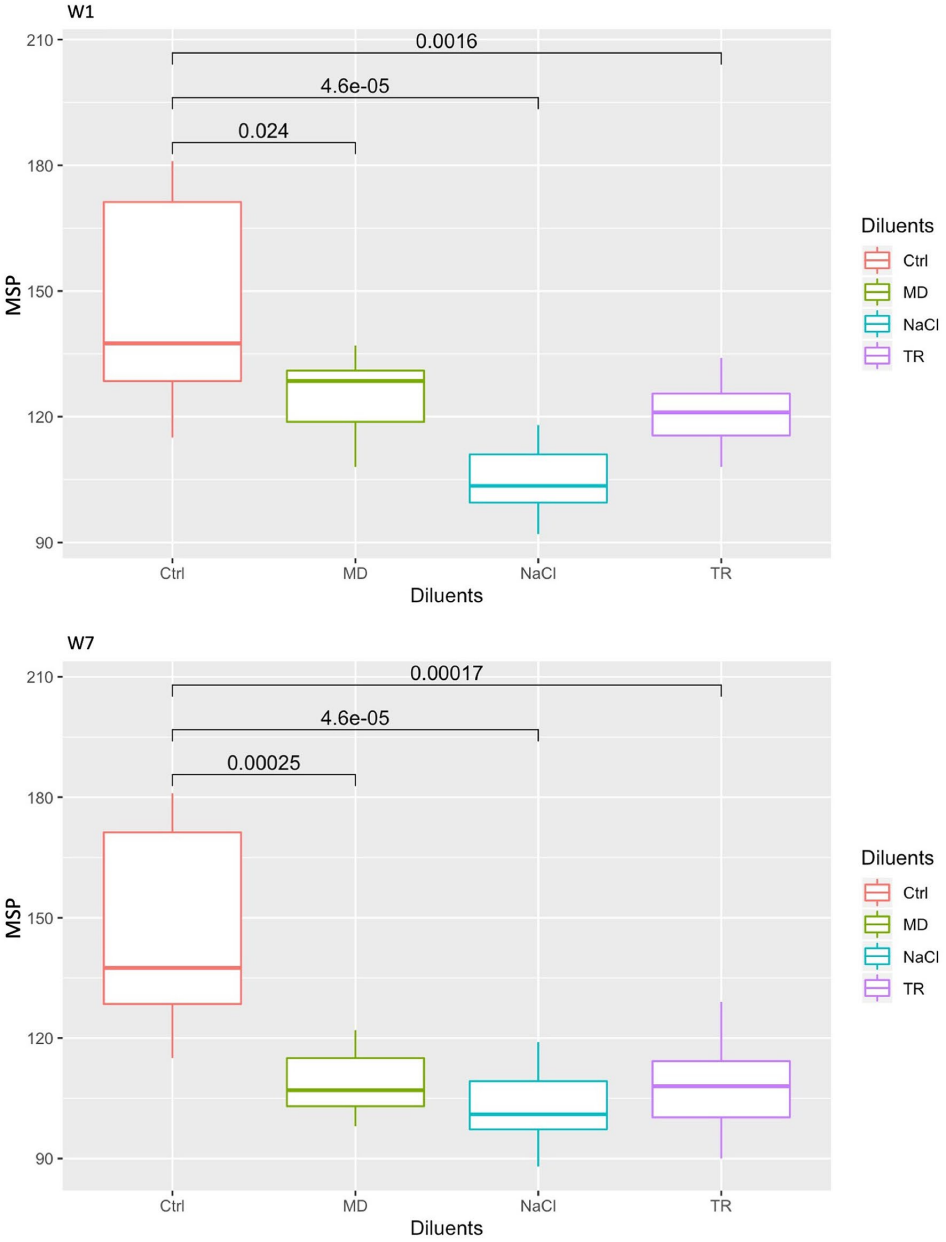
MSP richness in mice transplanted with either a fresh human stool specimen in NaCl (group Ctrl), or the same stool specimen that has been stored at − 80 °C for one week (top panel) or seven weeks (lower panel) in either NaCl, or MD, or TR. Analyses were performed on feces collected on day 2, 4 and 15.

We first compared the MSP richness in mice inoculated with different transplants, either freshly prepared or frozen in NaCl, MD or TR for one or seven weeks, by gathering MSP counts over two weeks following inoculation (day 2, 4 and 15, abbreviated D2, D4 and D15). As illustrated by Fig. 2, the global gut microbial MSP richness was significantly depressed in all groups of mice inoculated with the frozen transplants compared to control mice receiving the fresh preparation. However, the statistical significance was highest for mice inoculated with the frozen-thawed NaCl transplants after both one and seven weeks of frozen storage.

We then proceeded to a more refined analysis of the engraftment capacity of each experimental frozen transplant compared to the freshly prepared inoculum. For this purpose, we computed all pairwise Spearman correlations between experimental and control mice, taking into account the relative abundances of MSP in each fecal sample. We then performed a three-way mixed ANOVA to know how engraftment capacity of the frozen transplants, as assessed by correlations to controls, differed depending on the diluent (MD, TR, or NaCl) and the storage duration (W1 or W7), at the three observation times (D2, D4 and D15).

We found a significant three-way interaction between diluent, week of storage, and observation day (p.adj = 6.9e−03, Table 1), which means that at least one two-way interaction differed across the levels of the third independent variable. Therefore, we looked in more detail at the two-way interactions (Supplementary Information 1): week:day for each diluent, diluent:day for each storage duration, and week:diluent at each observation time. We found that the engraftment kinetics (day) depended on the storage duration (week) for the NaCl diluent only (p.adj = 2.7e−03), and depended on the diluent for a storage duration of seven weeks only (p.adj = 1.2e−02). The diluent effect depended on the storage duration at all observation times (all p.adj ≤ 3.2e−03).

**Table 1:** Results of the three-way mixed ANOVA. ges = generalized eta squared.

Effect	F	p	ges	p.adj	p.adj < .05
Diluents	131.485	6.97e−27	0.718	4.879e−26	*
Week	2.255	1.37e−01	0.021	9.590e−01	
Day	6.720	3.00e−03	0.013	2.100e−02	*
Diluents:Week	13.451	8.27e−06	0.207	5.789e−05	*
Diluents:Day	2.506	5.80e−02	0.010	4.060e−01	
Week:Day	0.654	4.88e−01	0.001	1.000e + 00	
Diluents:Week:Day	5.571	9.81e−04	0.021	6.867e−03	*

### Assessment of the diluent effect

**Figure 3:**
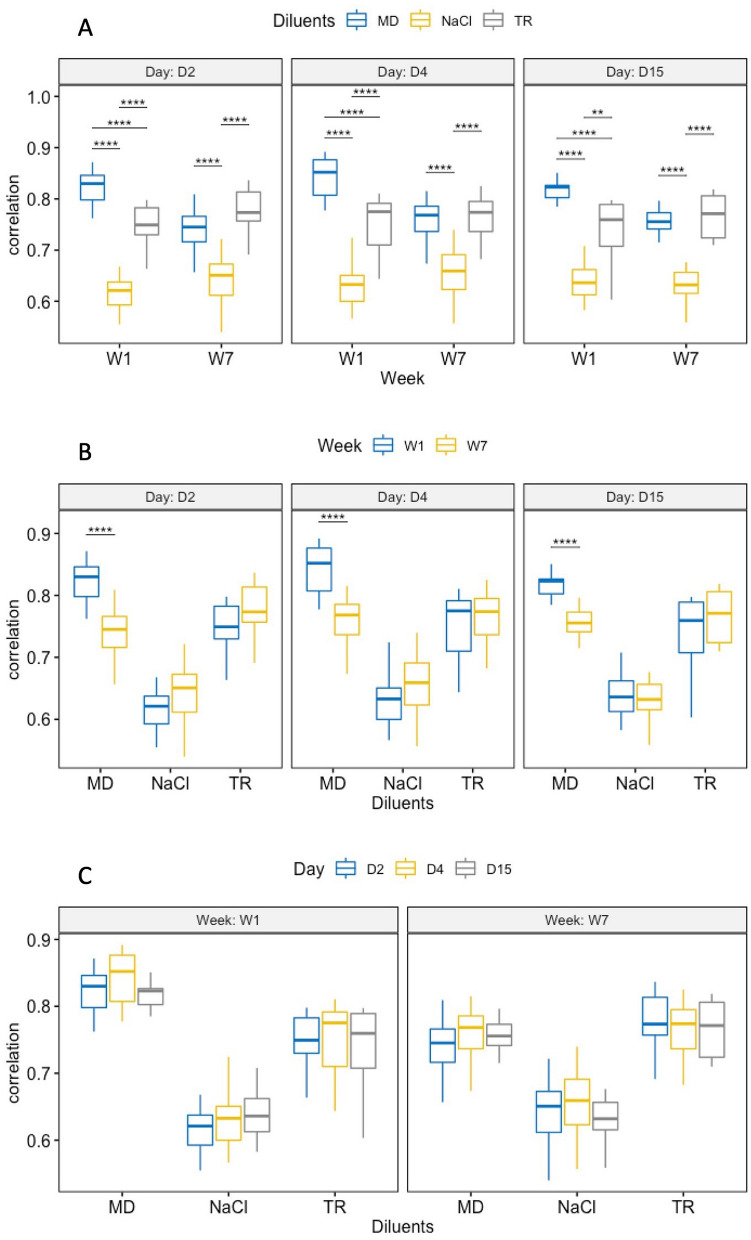
Pairwise comparisons between each cell of the design. (**A**) Effect of the diluent. (**B**) Effect of week of cryoconservation. (**C**) Day of observation.

We investigated the effect of the diluent on the engraftment capacity at every observation day and for each storage duration. There was a statistically significant simple main effect of diluent at all observation days and for both storage duration (all p.adj ≤ 1.5e−08, Supplementary Information 2). We computed all multiple pairwise comparisons to determine which diluents were different (Fig. 3A). After one week of storage, the MD transplant gave the MSP profiles that best resembled those of mice inoculated with the fresh inoculum (mean Spearman correlations of 0.83), while the NaCl transplant gave the most distant results (mean Spearman correlations of 0.63), and the TR transplant intermediate results (mean Spearman correlations of 0.74), and this was true at all three observation times (all p.adj ≤ 3.0e−03, Fig. 3A and Supplementary Information 3). Inoculation with the same transplants that had been frozen for six more weeks confirmed that MD and TR transplants gave better results than NaCl transplants, but there was no more superiority of MD over TR.

### Assessment of the storage duration effect

We then investigated the effect of the storage duration on the engraftment capacity at every observation day and for each diluent. There was a statistically significant simple main effect of storage duration only for the MD diluent at all observation days (all three p.adj ≤ 1.9e−04, Supplementary Information 2). Computation of all pairwise comparisons provided evidence that the high engraftment capacity of the MD transplants observed at W1 significantly decreased at W7 at the three observation times (all p.adj ≤ 3.3e−05, Fig. 3B and Supplementary Information 3), moving to levels of the TR transplants.

### Assessment of the engraftment kinetics

We finally investigated the engraftment kinetics at every storage duration and for each diluent. There was a small, but statistically significant simple main effect of the observation day for the NaCl diluent at W7 only (p.adj = 0.042, Supplementary Information 2). However, when computing all multiple pairwise comparisons, none of them was significant (Fig. 3C and Supplementary Information 3). As there was only a small main effect of the observation day, we continued with the multiple testing of differentially abundant MSP by grouping the data by diluent and storage duration, while gathering all observation times.

### Taxonomic analysis

**Figure 4:**
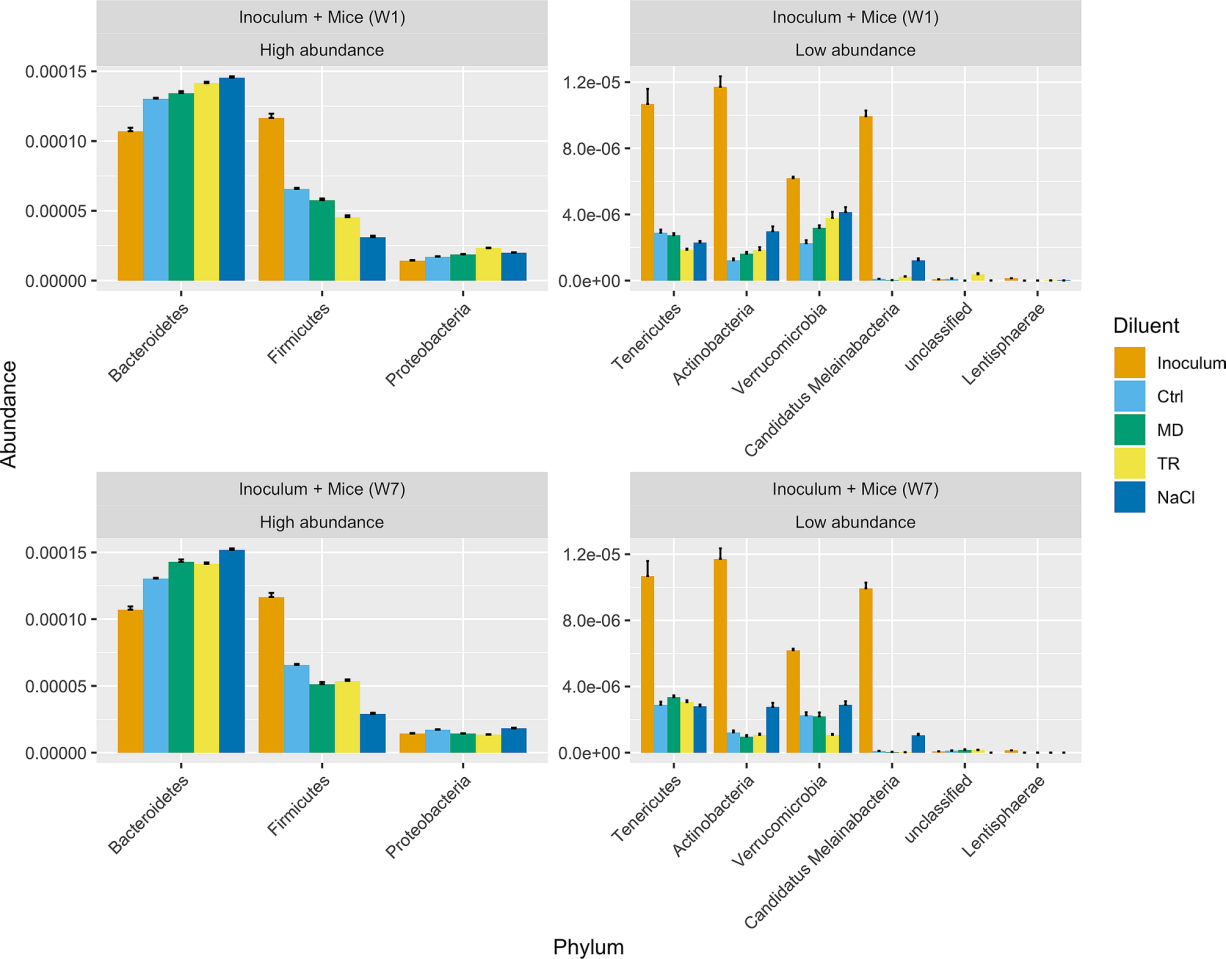
Total abundances of MSP at the phylum level in the inocula and the transplanted mice. Ctrl: mice transplanted with the fresh inoculum in NaCl; MD, TR, NaCl: mice transplanted with the same stool specimen that has been stored at − 80 °C for one week (top panels) or seven weeks (lower panels) in either MD, or TR, or NaCl. Values are means with standard errors in mice feces collected at day 2, 4 and 15, or in NaCl, MD and TR inocula.

We found the highest numbers of either proliferating or extinguishing MSP in mice inoculated with the frozen NaCl transplants, stored for either one or seven weeks (Supplementary Information 4 and 5, all comparisons against mice colonized with fresh transplants). The number of impacted MSP was far less numerous in mice inoculated with MD or TR than with NaCl frozen transplants (Supplementary Information 4 and 5). The change in abundance (q-value < 0.05 and log Fold Change >|2|) concerned 8% of the MSP for the MD transplant stored for one week (18% for seven weeks); 14% of the MSP for the TR transplant stored for one week (16% for seven weeks) and 30% of the MSP for the NaCl transplant stored for one week (24% for seven weeks). In all cases, extinguishing MSP were essentially from the family *Lachnospiraceae*, and to a lesser extent *Ruminococcaceae*, including *Faecalibacterium prausnitzii* whose species 1 and 3 appeared more exposed to extinction than species 4 (Supplementary Information 4, 5 and 6). However, total abundance of *Lachnospiraceae* was clearly depressed in mice receiving the defrosted NaCl transplants only (Supplementary Information 6 provides a comprehensive view of MSP abundances grouped by family and genus), which means that freezing in MD and TR was efficient in preserving many other species of this important family embedding a total of 91 MSP. Accordingly, richness observed within the families *Lachnospiraceae* and *Ruminococcaceae* was better preserved in frozen MD and TR transplant recipient mice compared to frozen NaCl transplant recipients (Supplementary Information 7). However, based on abundances, engraftment of these two taxonomic families, which was the highest in W1 frozen MD transplant recipients, was less remarkable in W7 frozen MD recipients, just as it was for the transplant taken as a whole (compare Supplementary Information 8 and Fig. 3B). In addition, fifteen to eleven MSP of diverse phylogenetic positioning, including a member of the family *Enterobacteriaceae*, increased in relative abundance in mice inoculated with frozen NaCl transplants compared to controls, while only a couple of MSP increased following inoculation with frozen MD or TR transplants (Supplementary Information 4-3 and 4-4). As a result, the overall abundance of Firmicutes members was close in mice receiving either the fresh transplants or those frozen in MD or TR, while it was half depressed in mice receiving the transplants frozen in NaCl (Fig. 4). However, in all cases, including mice receiving the fresh transplants, abundances of Firmicutes, Actinobacteria and other minor phyla were far depressed compared to that measured in the human inocula, while those of Bacteroidetes and Proteobacteria were increased (Fig. 4), which is a standard outcome in humanized germ-free mice^28–30^.

### Within-group consistency of the engraftment capacity

Interestingly, within-group Spearman correlations for MSP fingerprints showed that the engraftment capacity observed at D2, D4 and D15 was most reproducible in mice that received either the freshly prepared inoculum or the MD inoculum frozen for either one or seven weeks (Supplementary Information 9).

## Discussion

In a previous study^23^, we demonstrated that transplants prepared in maltodextrin-trehalose solutions, stored in a − 80 °C standard freezer and then rapidly thawed at 37 °C, retained a high revivification potential compared to transplants frozen in normal saline only, as proven by phylotranscriptomic profiles, metabolomic fingerprints, and flow cytometry assays over a 3-month observation period. Maltodextrin-trehalose based cryoprotectants were also very efficient in preserving viability of lyophilized transplants stored at ambient temperature^23^, an option that can be very attractive for fecal microbiota transplant biobanking and oral formulation. The lyophilized cakes were easily friable into a fine, light powder either beige colored when formulated with filtered crude fecal material, or white and odorless when using purified microbiota. The next step, herein reported, was to check the engraftment capacity of frozen/thawed liquid formulations of those novel ready-to-use human transplants, in germ-free born mice. Based on deep metagenomic shotgun sequencing of the fecal DNA extracted at three-time points (days 2, 4 and 15) following a single inoculation, we clearly observed that gavage with thawed MD or TR suspensions gave the MSP profiles that best resembled those obtained with the fresh inoculum, while engraftment capacity of thawed NaCl transplants most diverged. This was true at each observation time, and whether the conservation period was one or seven weeks. Although we cannot extrapolate engraftment capacities over longer periods of freezing, we previously demonstrated that the freezing–thawing cycle rather than duration of cryoconservation at − 80 °C, was the main cause for the loss of bacterial viability in fecal microbiota transplants^23^. It is then hardly surprising that frozen-thawed MD and TR transplants did efficiently colonize the mouse gut, whether they were kept frozen for one or seven weeks. Human transplants freeze-dried in either 5% trehalose and stored at − 80 °C to avoid rehydration^28^, or in a maltodextrin/trehalose cocktail and simply stored at 4 °C for one year^30^, have further been shown to colonize the mouse gut as efficiently as suspensions frozen in glycerol^28,30^, and to successfully increase survival to experimental *Clotridium difficile* infection^30^, even though the genetic background of mice do not allow to reproduce the profile of the human commensal microbiota^29^.

Engraftment of members of the family *Lachnospiraceae*, and to a lesser extent *Ruminococcaceae*, including members of the genus *Faecalibacterium*, was the most challenging in all experimental groups, being improved compared with NaCl but still depressed compared with controls, in mice inoculated with frozen MD and TR transplants. This coincides with our previous observations indicating that members of these families make part of the most challenging commensals for revivification of transplants in culture^23^. Improvement of revivification and engraftment of these important butyrate-producing groups in maintaining health^31,32^ represents an interesting issue that could benefit from further research on fecal microbiota transplant manufacturing. This is all the more important since abundance of families *Lachnospiraceae* and *Ruminococcaceae* is believed to be a good predictor of FMT success in the recipient^13,33^, particularly in *C. difficile* infected patients^34,35^, while engraftment of these families in the FMT recipient is not straightforward^36^. In this respect, mouse models can be used advantageously in a first approach since one mouse enterotype is dominated by *Lachnospiraceae*/ *Ruminococcaceae*, similarly to the human *Firmicutes* enterotype (also known as enterotype 3)^37^. Another interesting result deduced from pairwise comparisons between mice within the same group, was the higher consistency of engraftment of the thawed MD transplants compared to thawed TR or NaCl transplants. Even though we do not have a good explanation for this, nor the certainty that the same would apply to humans, one could prefer MD transplants to TR transplants for their consistency to colonize the gut of any recipients, provided that engraftment capacity of MD-preserved transplants was maintained over extended storage times. That the highest engraftment capacity of W1 frozen MD transplants could have been shifted to less remarkable levels after seven weeks of storage could not be anticipated and remains unexplained, given that we previously demonstrated that the freezing–thawing cycle rather than the duration of cryopreservation, was the main cause for the loss of bacterial viability assessed by culture and flow cytometry in diverse transplants (prepared in either MD, or TR, or NaCl, and from different donors), over a 3-month observation period^23^. At last, within group pairwise correlations decreased as time elapsed after inoculation in all groups, indicating that remodeling of the inoculum occurred in part independently of species-specific and genetic factors in those mice of the same strain, with a likely impact of individual behavioral patterns.

## Methods

### Donor information and ethics

A healthy human donor with neither symptoms nor a family history of gastrointestinal disorder and with no use of antibiotics within the preceding two months was asked to provide a single fecal sample that was procured, collected, stored and disseminated in accordance with the highest ethical standards and in strictest compliance with all applicable rules and regulations. This included ensuring that informed consent was fully informative to the donor and that the donor's wishes in relation to the use of the sample were strictly complied with. The study was approved by the ethics committee CPP of Ile de France 1 under number 13642.

### Animals

The FMT experiment involved twenty-eight 8-week-old germ-free male mice, strain C57BL/6 J, produced, bred and experimented in the animal facilities of ANAXEM (Micalis Institute, INRA Jouy-en-Josas, France). They were housed in individual cages, which were themselves installed in seven gnotobiotic isolators, one per treatment group (see below), in a facility approved by the French Department of Veterinary Management. Housing and all experimental procedures were in compliance with the European legislation, with the ARRIVE guidelines and had been approved by the local ethics committee (registered under the number 2015060510305108_v3, APAFISH#775). All mice were given free access to autoclaved tap water and γ-irradiated (45 kGy) standard pelleted chow (SAFE R03/4).

### Experimental design

Two novel, glycerol-free diluents (referred as MD and TR for maltodextrin:trehalose, 3:1, w/w, and trehalose:maltodextrin 3:1, w/w, respectively) were used to prepare and keep frozen experimental fecal microbiota transplants of human origin. These formulations were compared to a saline suspension (referred to as NaCl) for their efficiency to colonize the germ-free mice. A single human fecal sample was re-suspended in the appropriate diluent (MD, TR or NaCl) and stored for one or seven weeks prior to being used for inoculation of mice. The reference/control inoculum (referred to as CTRL) was the stool dilution freshly prepared in NaCl immediately prior to inoculation. All FMTs were carried out by oral gavage of a single dose of 200 µL of fecal suspension (about 10^10^ bacteria) plus four to five drops on the litter*.* One group of four mice, used as controls, received the transplant freshly prepared in NaCl. One week later, three other groups of four mice were each inoculated with one of the three transplants in either NaCl, or MD or TR that had been kept at − 80 °C for the week. Lastly, three additional groups of four mice were each inoculated with the same three transplants that had been frozen for six more weeks. Feces of all mice were individually collected at three-time points: on the second, 4th and 15th days following inoculation. No loss of animals or undesired events were registered during the experiment. Mice were all raised in individual cages.

### Transplant preparation and use

The protocol included the following diluents: physiologic saline 9 g/L NaCl (27810.295 from VWR), identified ‘NaCl’ ; maltodextrin DE 6 (GLUCIDEX® Maltodextrin 6 from Roquette) 18.75% (w/v) + trehalose (90210-250G from Fluka) 6.25% (w/v) in saline 9 g/L, identified ‘MD’ ; trehalose 18.75% + maltodextrin 6.25% in saline 9 g/L, identified ‘TR’. The diluents were filter-sterilized on PES membrane 0.22 µm, degassed in a 90 °C water bath for 30 min, and then allowed to stand at ambient temperature (20 ± 2 °C) in an anaerobic Freter chamber (90% N2, 5% H2 and 5% CO2) for 48 h before use. The stool sample was transferred to the anaerobic chamber within 2 h after collection. Aliquots of stool were weighted in Stomacher Filter Bags (Seward BA6041/8TR, or VWR 432–3119, 0.5 mm holes). The appropriate volume of diluent was added (i.e. 4 mL of diluent per g of stool, final concentration of MD and TR, 15 and 5%, respectively for the MD transplants, or the reverse for the TR transplants). All suspensions were further supplemented with the two reducing agents, sodium L-ascorbate and L-cysteine hydrochloride monohydrate, to a final concentration of 5% (w/v) and 0,1% (w/v), respectively. A 5-min hand mixing throughout the bag ensured both homogenization and filtration. Two 2-mL fractions of each filtered fecal suspension were transferred into CryoTubes and frozen at − 80 °C until use for inoculation, while fresh fractions of the NaCl suspension was immediately used for inoculation. When used after cryopreservation, the suspensions were rapidly thawed in a water bath at 37 °C prior to inoculation.

### Metagenomic profiling

Total DNA was extracted from animal fecal pellets or inoculated preparations by the SAMBO MGP platform using both physical and chemical lysis following standard operating procedures (IHMS_SOP 06 available at http://www.microbiome-standards.org/index.php?id=253). DNA was quantitated using Qubit Fluorometric Quantitation (ThermoFisher Scientific, Waltham, US) and qualified using DNA size profiling on a Fragment Analyzer (Agilent Technologies, Santa Clara, US). Thereafter all stool DNA preparations were subjected to shotgun metagenomic sequencing using the Ion Proton Sequencer (Thermo Fisher Scientific). Sequencing data were processed using the METEOR software suite (software available upon request). First, high quality reads were selected and cleaned with AlienTrimmer^38^ and filtered by average quality threshold to eliminate sequencing adapters, contaminants and low quality reads. HQ clean reads were then mapped against the updated Integrated Gene Catalogue of the human gut microbiome (IGC2, 10.4 million genes)^39^ using Bowtie2. Alignments with identity ≥ 95% were kept to account for gene variability and the non-redundant nature of the catalogue. Gene counts were computed with a two-step procedure as previously described^40^. To decrease technical bias due to different sequencing depth and avoid any artifacts of sample size on low-abundance genes, read counts were ‘rarefied’. The gene abundance table was rarefied to 12 M mapped reads (a threshold chosen to include all samples) using a random sampling procedure without replacement. The resulting rarefied gene abundance table was normalized according to the FPKM strategy (normalization by the gene length and the number of total mapped reads reported in frequency) to give the final gene abundance profile table. This table was used as a starting point to perform the analysis using the suite of R packages MetaOMineR developed at MGP^41^, and dedicated to the analysis of large quantitative metagenomics datasets.

### Metagenomic species

The IGC2 gene catalogue has been clustered into 1990 Metagenomic Species Pangenome (a MSP is a cluster of co-abundant genes belonging to the same microbial species computed among 1601 samples) using MSPminer^42^. Taxonomic annotation of each MSP was performed using all core and accessory genes by sequence similarity searches against previously sequenced organisms in NCBI using BLASTn against WGS database (restricted to Bacteria, Archae, Fungi, Viruses and Blastocystis) and nt database (restricted to Bacteria, Archae, Fungi, Viruses, Homo sapiens, Bos, Arabidopsis thaliana, Gallus gallus and Sus scrofa. A species-level assignment was given if > 50% of the genes matched the same reference genome of the NCBI database (September 2018 version) at a threshold of 95% of identity and 90% of gene length coverage. The remaining MSP were assigned to a given taxonomic level from the genus to the superkingdom level, if more than 50% of their genes had the same level of assignment, or according to their position in the phylogenetic tree^42^. Abundance profiles of each MSP were computed as the mean abundance of their 50 best core genes.

### Statistical analyses

We used the Spearman’s correlation coefficient to assess the similarity between the abundance profiles of MSP across samples. A three-way mixed ANOVA was performed to evaluate the effects of storage duration, diluents and time on transplant engraftment. It is used to evaluate if there is a three-way interaction between three independent variables, including two between-subject (Week W1/W7, Diluents MD/TR/NaCl) and one within-subject factors (days D2/D4/D15). The ANOVA assumptions have been checked: there were no extreme outliers, as assessed by box plot method. The data were normally distributed, as assessed by inspection of the QQ plot. There was homogeneity of variances (*p* > 0.05) as assessed by Levene’s test at each level of the within-subjects factor (time), except for D15 (p = 0.002). However, ANOVA is quite robust to heterogeneity of variance when the sample sizes are equal. There was homogeneity of covariances of the between-subject factors (storage duration and diluents), as assessed by Box’s M-test (*p* > 0.001). For the three-way interaction effect, Mauchly’s test of sphericity indicated that the assumption of sphericity was not met (*p* < 0.05), and the Greenhouse–Geisser sphericity correction has been applied. P-values have been adjusted using the Bonferroni multiple testing correction method, using a significance threshold of 5%.

Search for the significantly impacted taxa was carried out using the Wilcoxon test against fresh NaCl all time samples (Ctrl) against frozen samples NaCl all time / TR all time / MD all time (samples frozen for one week on one side, for seven weeks on the other side). When performing multiple tests, the Type I error rate tends to become inflated. The Benjamini–Hochberg adjustment was used to control the False Discovery Rate (FDR), using a significance threshold of 5%.

## Supplementary Information

Supplementary Information 1–3.

Supplementary Information 4, 6–9.

Supplementary Information 5.

## Data Availability

Raw sequencing data used in this study have been deposited in the EMBL-EBI European Nucleotide Archive (ENA) under accession number PRJEB39960.
