# Supplementary material for: High engraftment capacity of frozen ready-to-use human fecal microbiota transplants assessed in germ-free mice
Source: Sci Rep. 2021 Feb 23;11:4365. doi: 10.1038/s41598-021-83638-7 (PMC7902644; doi:10.1038/s41598-021-83638-7)
Supplement: Supplementary file 2 — Supplementary Information 4, 6–9. [file 41598_2021_83638_MOESM2_ESM.pdf]

High engraftment capacity of frozen ready-to-use human fecal transplants assessed in germ-free mice

Magali Berland[a]*, Julie Cadiou[b], Florence Levenez[a], Nathalie Galleron[a], Benoît Quinquis[a], Florence Thirion[a], Franck Gauthier[a], Emmanuelle Le Chatelier[a], Florian Plaza Oñate[a], Carole Schwintner[c], Sylvie Rabot[b], Patricia Lepage[b], Dusko Ehrlich[a], Joël Doré[a,b] and Catherine Juste[a,b]

[a]*Université Paris-Saclay, INRAE, MGP, 78350 Jouy-en-Josas, France ;*

[b]*Université Paris-Saclay, INRAE, AgroParisTech, Micalis Institute, 78350, Jouy-en-Josas, France;* [c]*MaaT Pharma, Pharmaceutical Development, 69007, Lyon, France*

*Magali Berland magali.berland@inrae.fr, INRAE, MGP, 78350 Jouy-en-Josas, France

W1

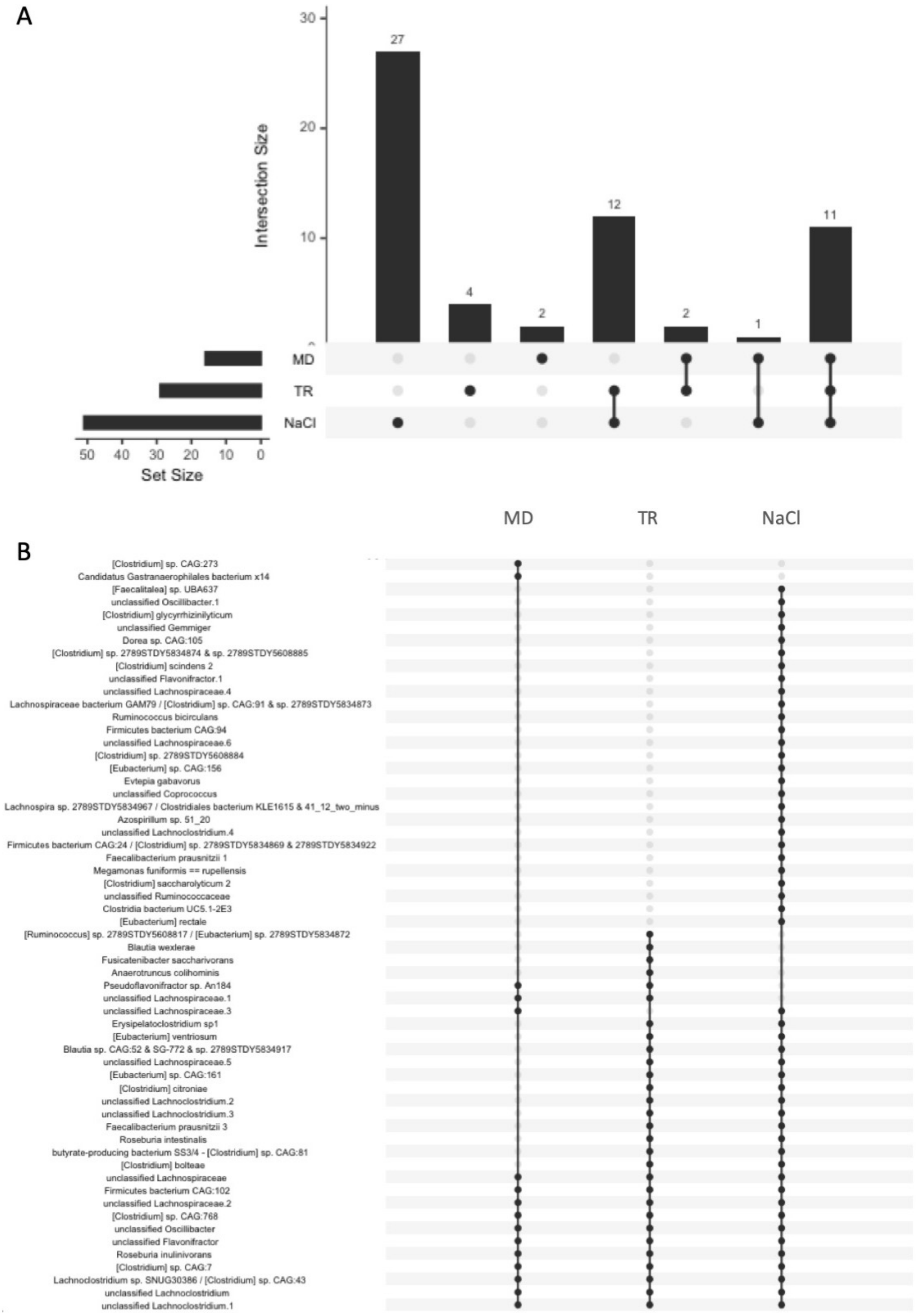

Supplementary Information 4-1. (A) UpSetR plot of the number of MSP whose abundance significantly decreased in mice inoculated with frozen compared to fresh transplants after one week of storage (q-value < 0.05 and log Fold Change > 2). The main bar chart on the top shows the number of MSP that specifically decreased in each experimental group or a combination of them, the black dots beneath indicating the combination. The smaller bar chart to the left shows the total number of MSP that decreased in each experimental group. (B) The list of extinguishing MSP for each diluent, annotated at the species level. Statistics and taxonomy of significantly affected MSP are detailed in Supplementary Information 5. All comparisons are against the control group inoculated with the fresh fecal transplant in NaCl.

W7

A

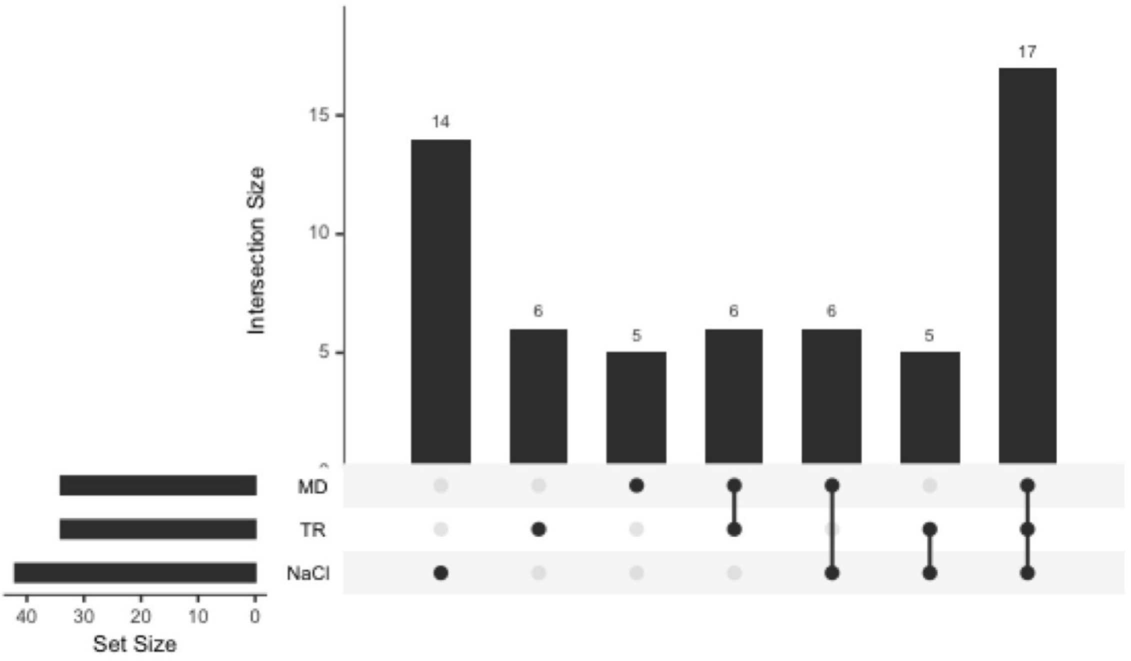

B

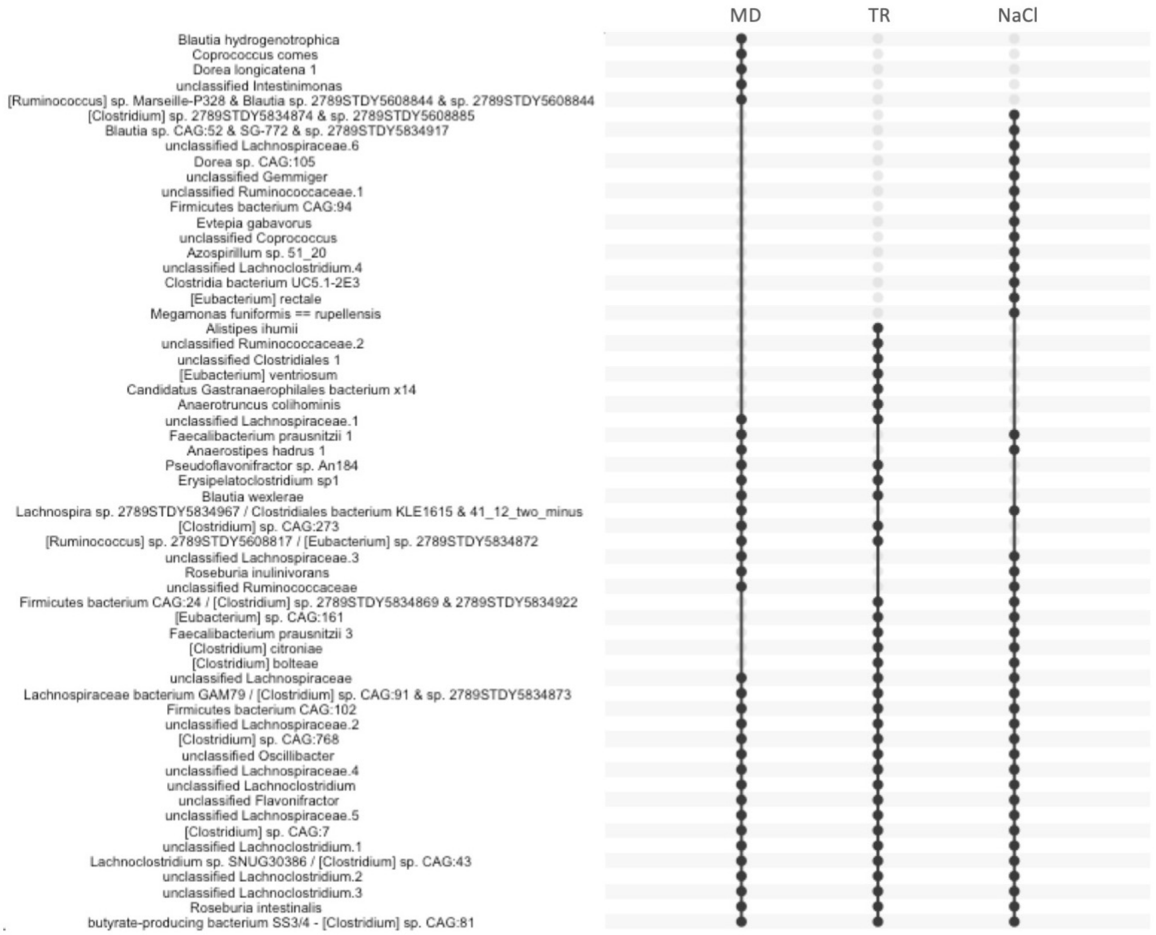

Supplementary Information 4-2. (A) UpSetR plot of the number of MSP whose abundance significantly decreased in mice inoculated with frozen compared to fresh transplants after seven weeks of storage (q-value < 0.05 and log Fold Change > 2). Reading of the graph is the same as for Supplementary Information 4-1. (B) The list of extinguishing MSP for each diluent, annotated at the species level. Statistics and taxonomy of significantly affected MSP are detailed in Supplementary Information 5. All comparisons are against the control group inoculated with the fresh fecal transplant in NaCl.

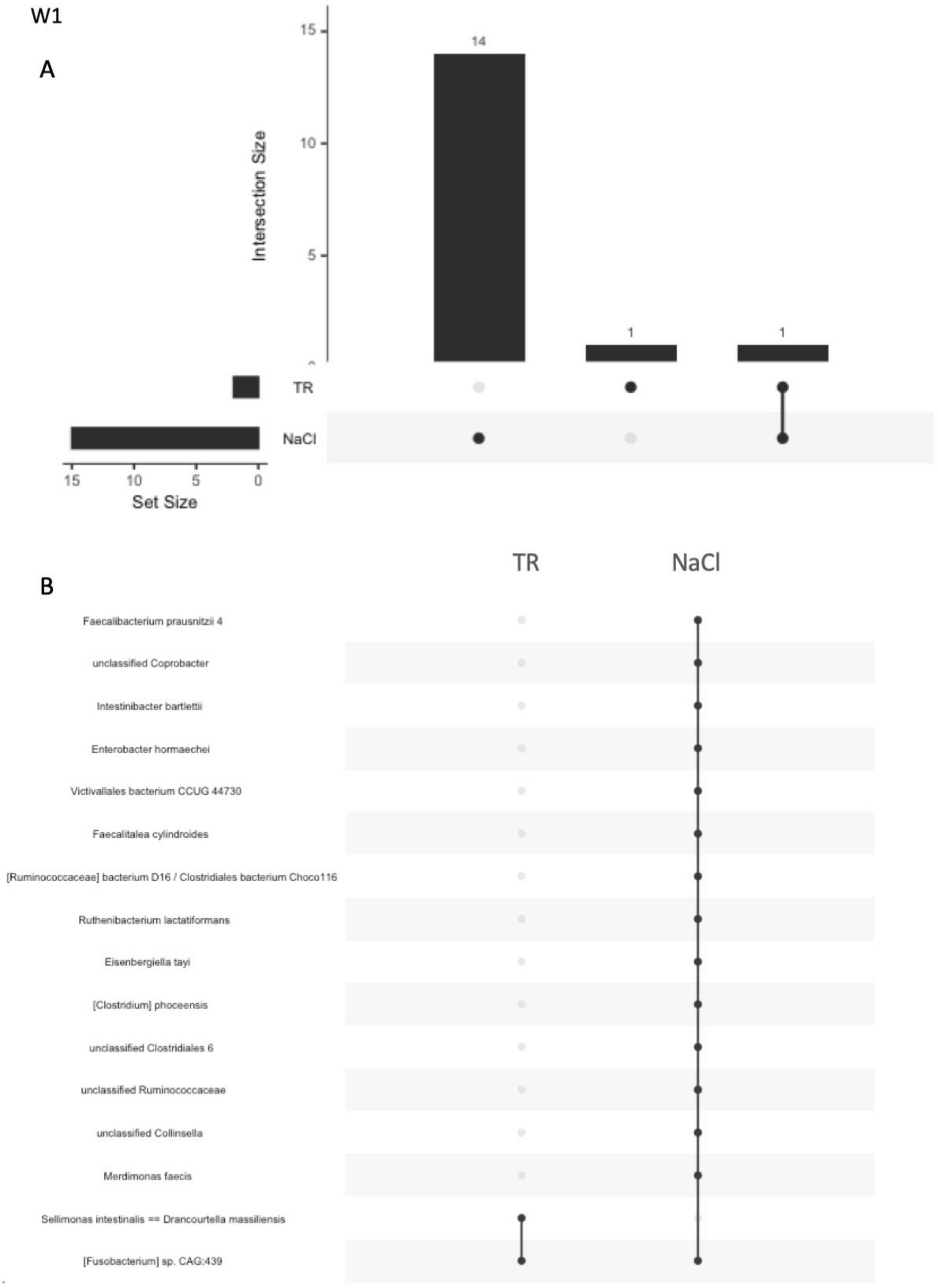

Supplementary Information 4-3. (A) UpSetR plot of the number of MSP whose abundance significantly increased in mice inoculated with frozen compared to fresh transplants after one week of storage (q-value < 0.05 and log Fold Change > 2). Reading of the graph is the same as for Supplementary Information 4-1. (B) The list of proliferating MSP for each diluent, annotated at the species level. Statistics and taxonomy of significantly affected MSP are detailed in Supplementary Information 5. All comparisons are against the control group inoculated with the fresh fecal transplant in NaCl.

W7

A

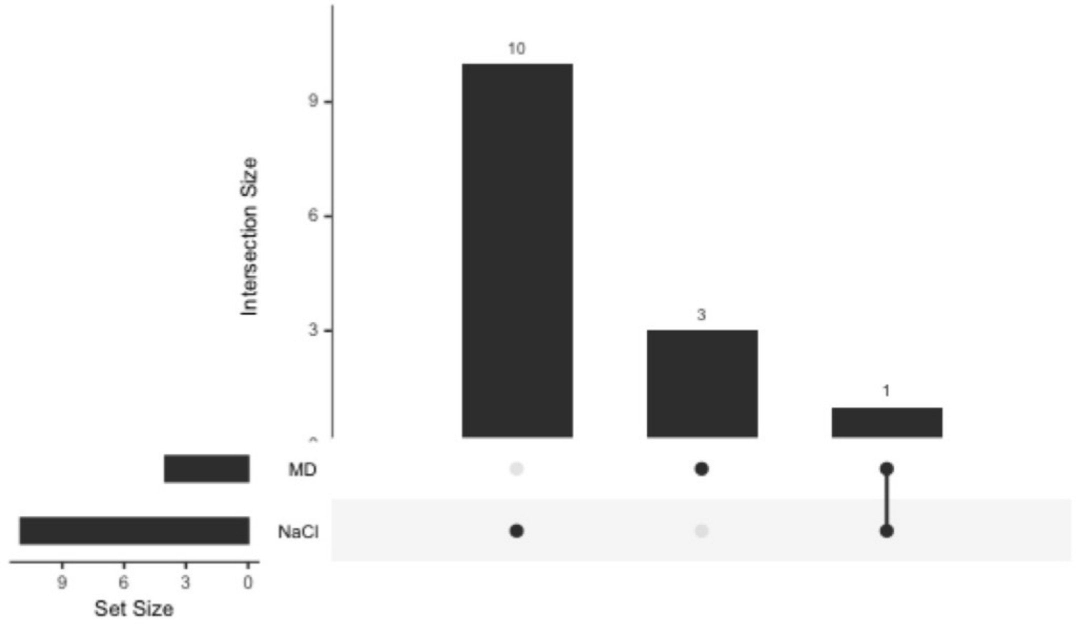

B

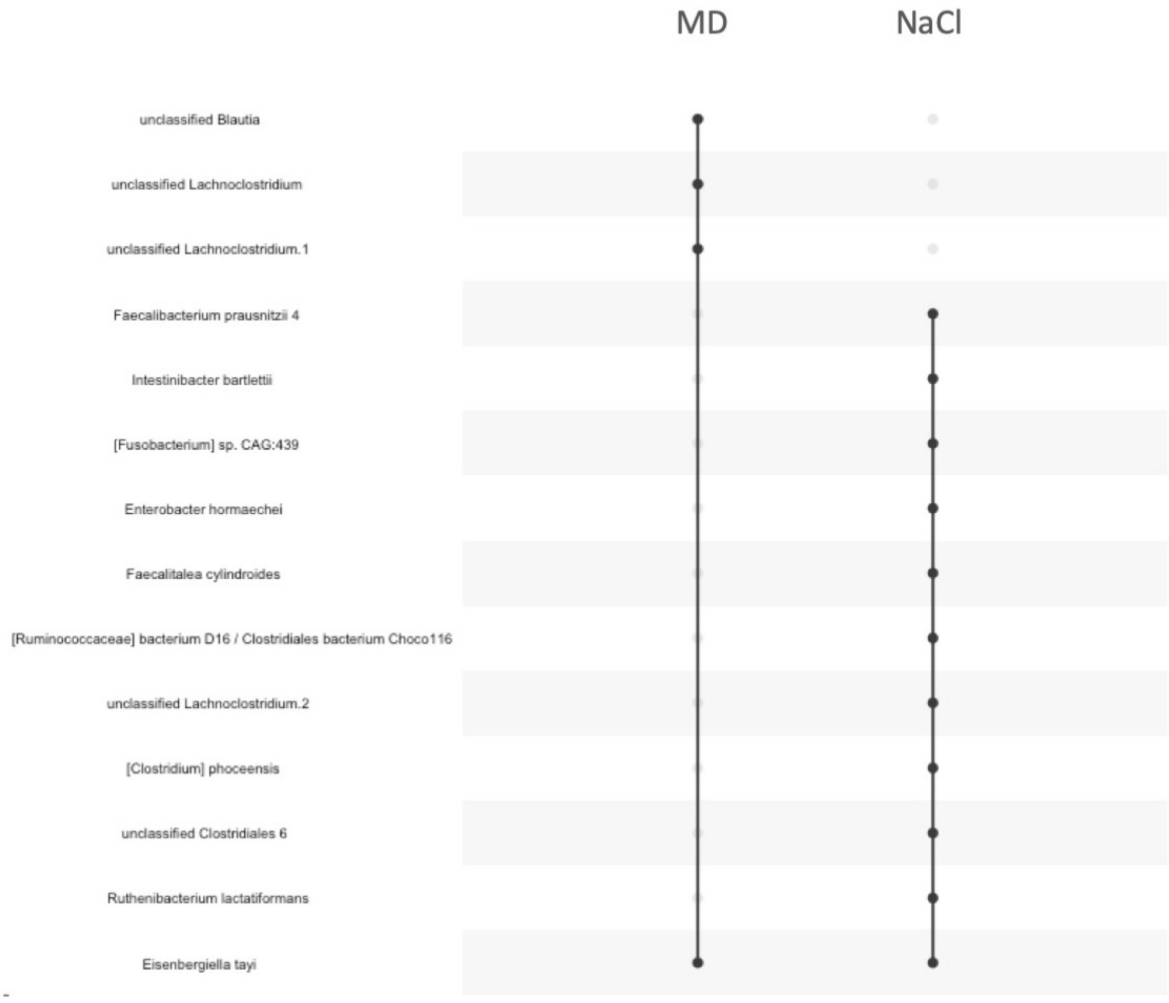

Supplementary Information 4-4. (A) UpSetR plot of the number of MSP whose abundance significantly increased in mice inoculated with frozen compared to fresh transplants after seven weeks of storage (q-value < 0.05 and log Fold Change > 2). Reading of the graph is the same as for Supplementary Information 4-1. (B) The list of proliferating MSP for each diluent, annotated at the species level. Statistics and taxonomy of significantly affected MSP are detailed in Supplementary Information 5. All comparisons are against the control group inoculated with the fresh fecal transplant in NaCl.

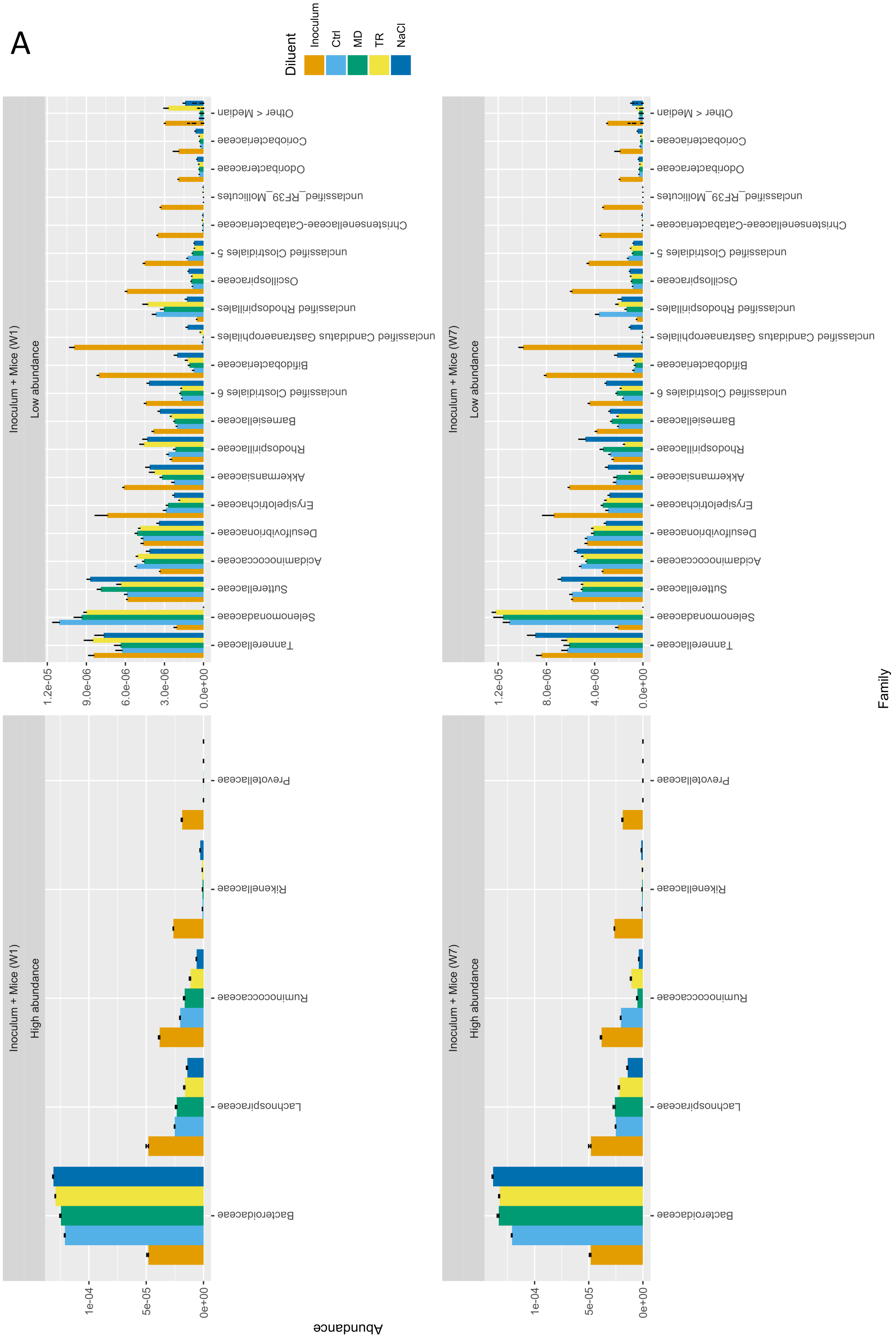

Supplementary Information 6. Total abundances of MSPs at the family (A) and the genus (B) levels in the inocula and the transplanted mice. Ctrl: mice transplanted with the fresh inoculum in NaCl; MD, TR, NaCl: mice transplanted with the same stool specimen that has been stored at -80 °C for one week (top panels) or seven weeks (lower panels) in either MD, or TR, or NaCL. Values are means with standard errors in mice feces collected at day 2, 4 and 15, or in NaCL, MD and TR inocula.

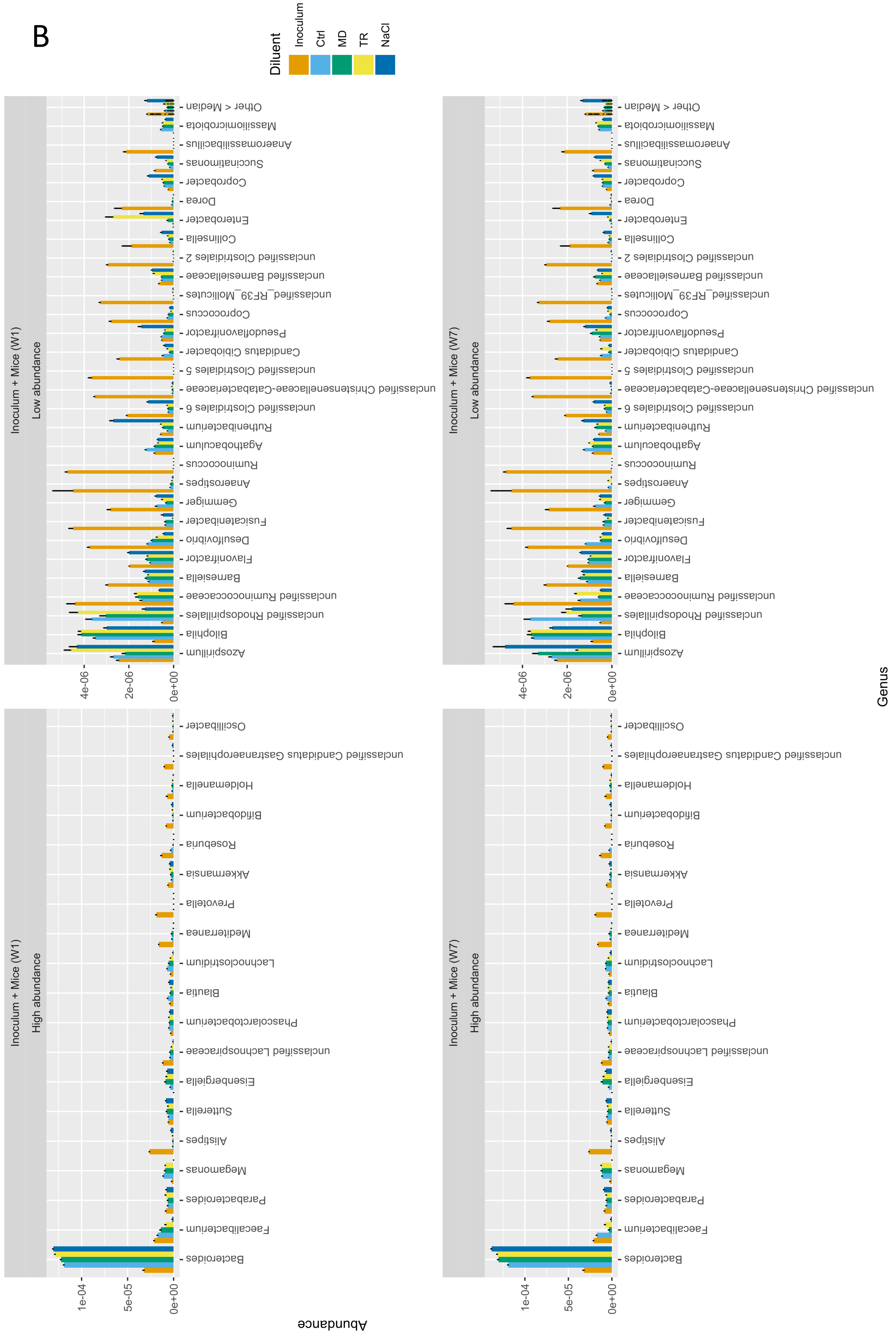

Supplementary Information 6. Total abundances of MSPs at the family (A) and the genus (B) levels in the inocula and the transplanted mice. Ctrl: mice transplanted with the fresh inoculum in NaCl; MD, TR, NaCl: mice transplanted with the same stool specimen that has been stored at -80 °C for one week (top panels) or seven weeks (lower panels) in either MD, or TR, or NaCL. Values are means with standard errors in mice feces collected at day 2, 4 and 15, or in NaCL, MD and TR inocula.

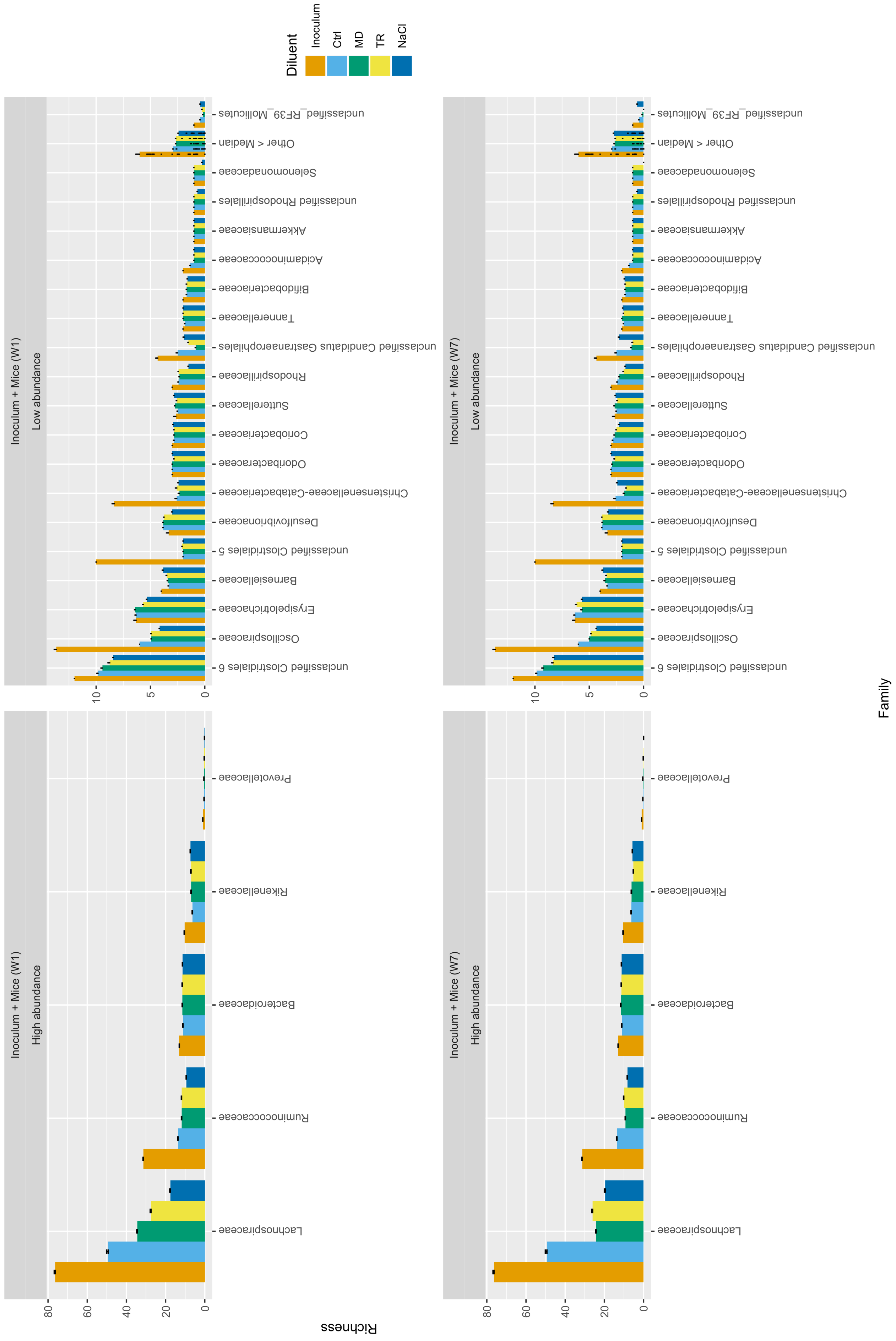

Supplementary Information 7. Total richness of MSPs at the family level in the inocula and the transplanted mice. Ctrl: mice transplanted with the fresh inoculum in NaCl; MD, TR, NaCl: mice transplanted with the same stool specimen that has been stored at -80 °C for one week (top panels) or seven weeks (lower panels) in either MD, or TR, or NaCl. Values are means with standard errors in mice feces collected at day 2, 4 and 15, or in NaCl, MD and TR inocula.

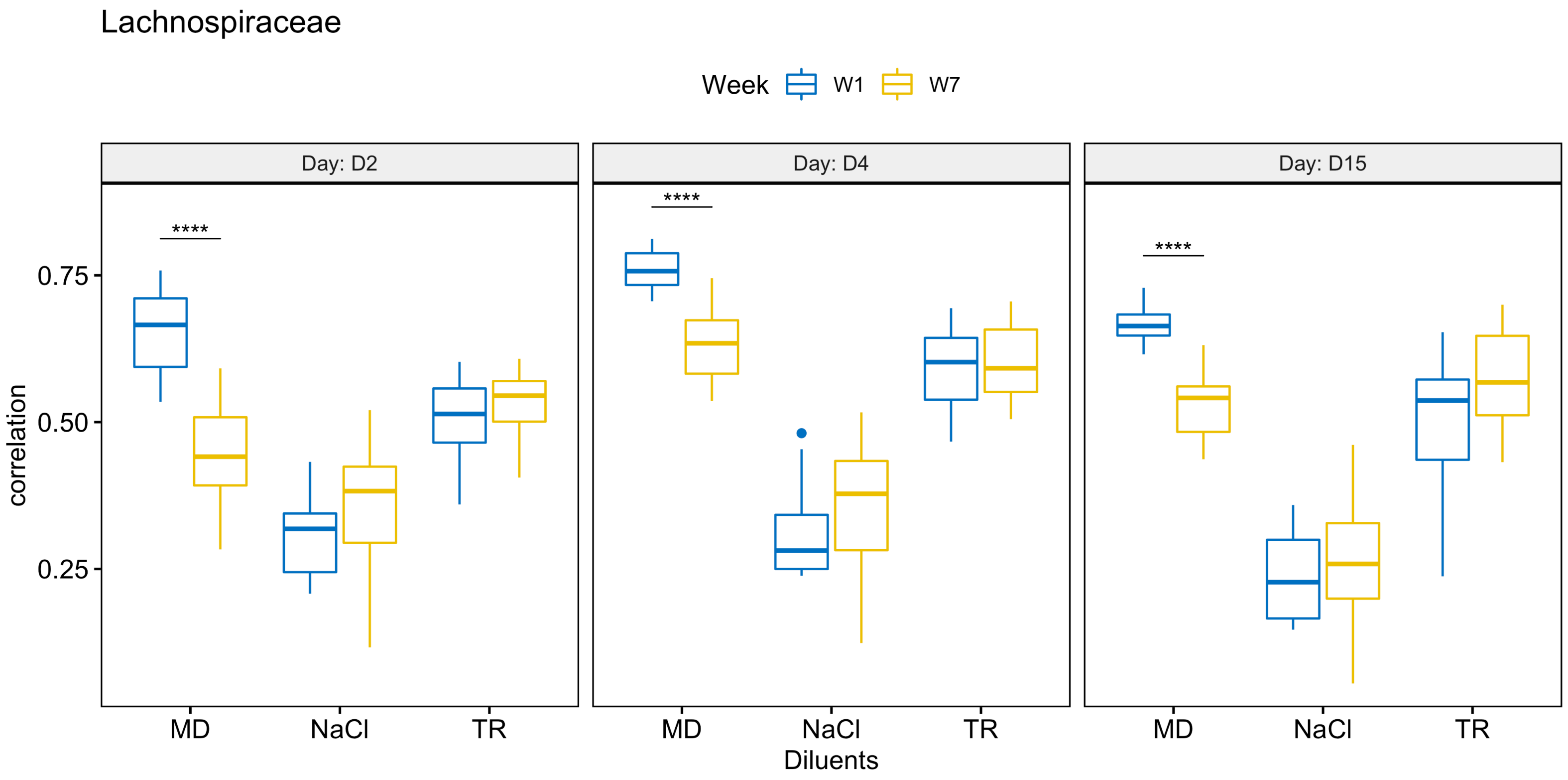

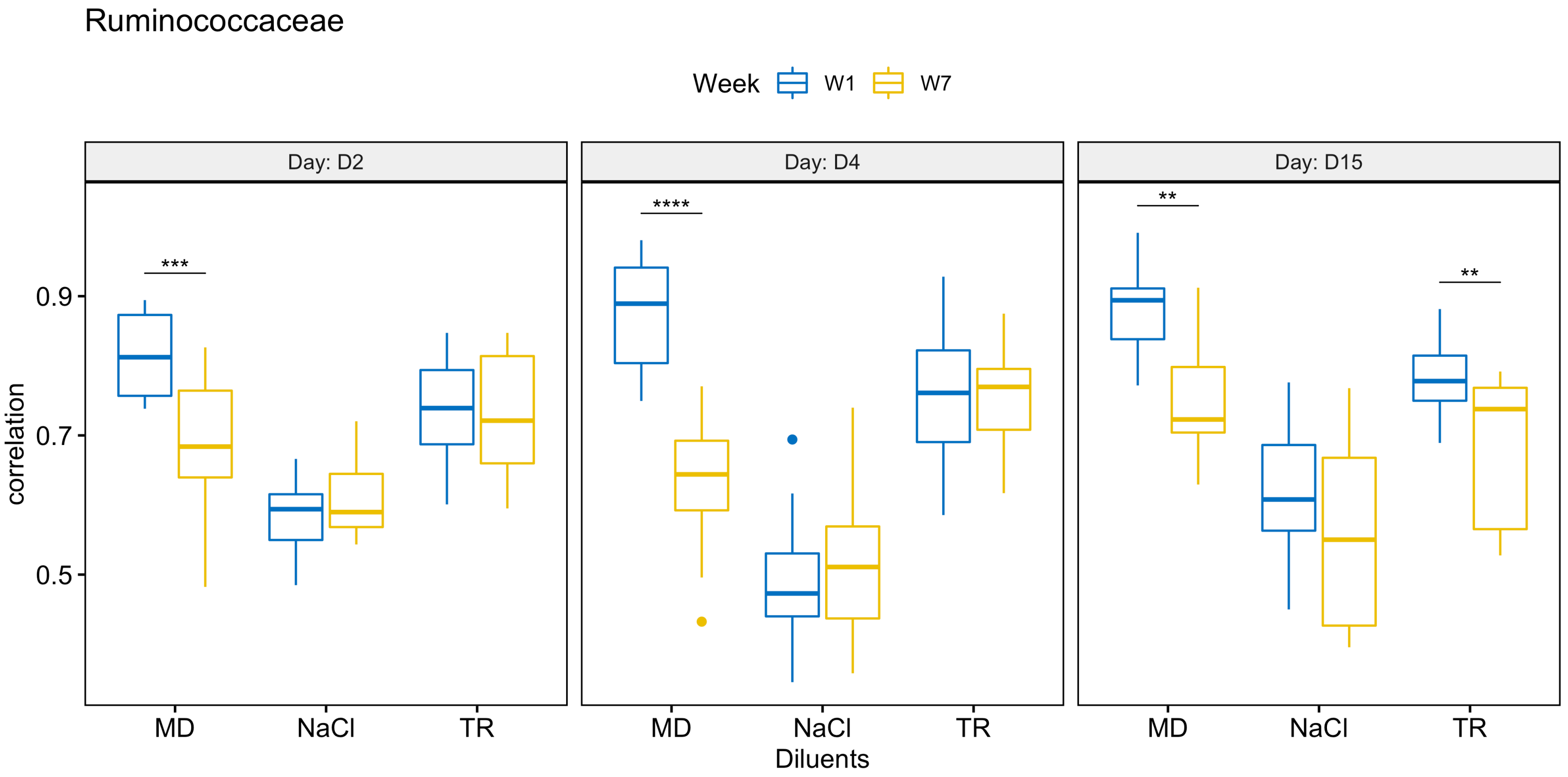

Supplementary Information 8. Decomposition of the Figure 3B for the Lachnospiraceae and Ruminococcaceae families: pairwise comparisons of the spearman correlations between Ctrl and each diluent.

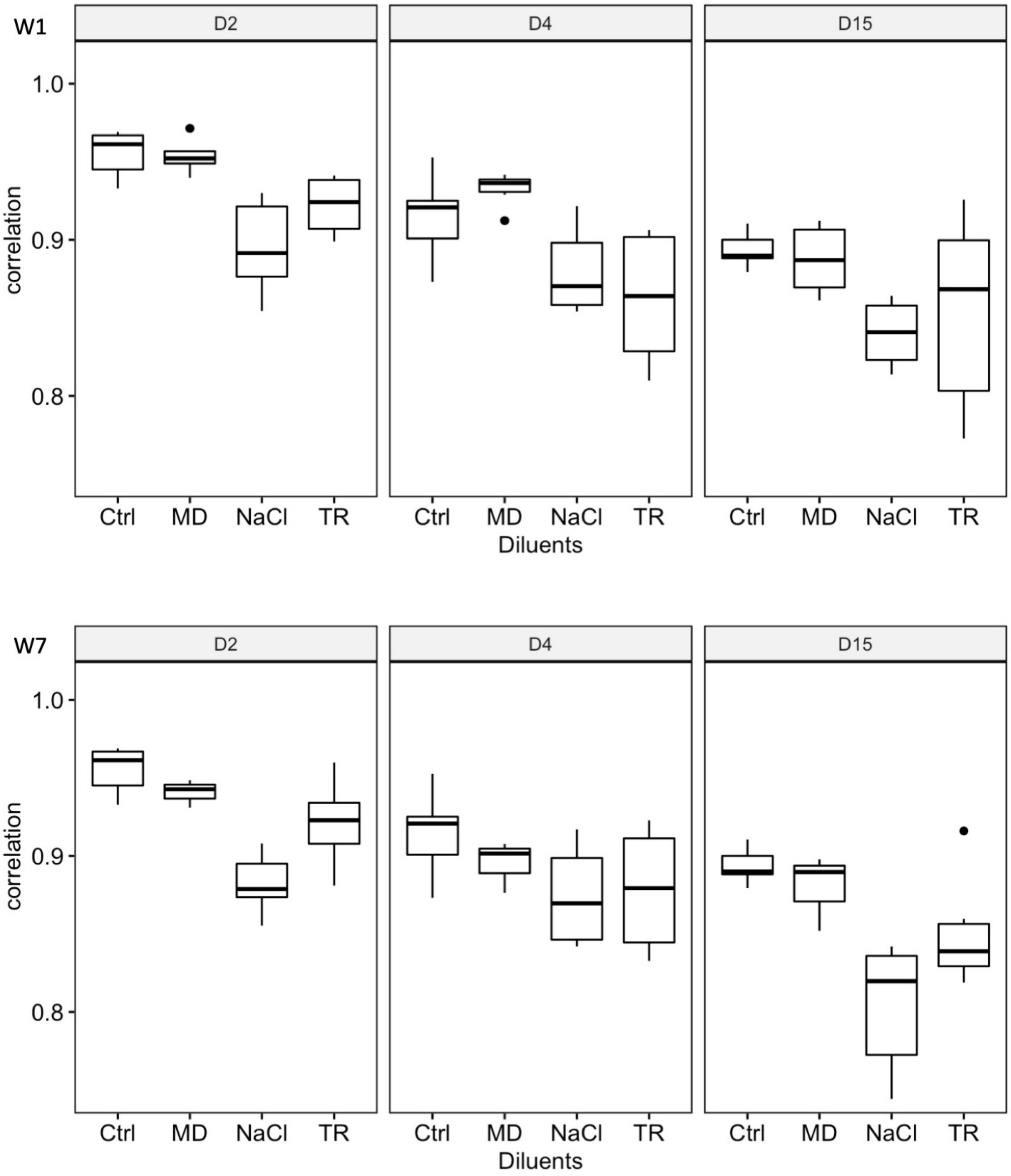

Supplementary Information 9. Within-group Spearman correlations for MSP fingerprints. Mice were transplanted with either a fresh human stool specimen in NaCl (group Ctrl), or the same stool specimen that has been preserved for one week (top panel) or seven weeks (lower panel) in either NaCl, or MD, or TR. Analyses were performed on feces collected at day 2, 4 and 15 (D2, D4, D15) following transplantation.
